# Functional results after hiatal repair and gastropexy without fundoplication in patients with paraoesophageal hernia

**DOI:** 10.1007/s00423-024-03340-w

**Published:** 2024-05-03

**Authors:** Laura Bomio-Pacciorini, Sérgio Gaspar-Figueiredo, Styliani Mantziari, Sébastien Godat, Markus Schäfer, Hugo Teixeira Farinha

**Affiliations:** 1 Department of Visceral Surgery, Faculty of Biology and Medicine UNIL, Lausanne University Hospital (CHUV), Rue du Bugnon 46, 1011 Lausanne, Switzerland; 2 Department of Gastroenterology and Hepatology, Faculty of Biology and Medicine UNIL, Lausanne University Hospital (CHUV), Rue du Bugnon 46, 1011 Lausanne, Switzerland

**Keywords:** Gastropexy, Paraoesophageal Hernia, Hiatal Hernia, Gastroesophageal reflux disease (GERD), Quality-of-life (QoL)

## Abstract

**Purpose:**

Paraoesophageal hernias (PEH) are associated with a high complication rate and often occur in elderly and fragile patients. Surgical gastropexy without fundoplication is an accepted alternative procedure; however, outcomes and functional results are rarely described. Our study aims to evaluate short-term outcomes and the long-term quality of life after gastropexy as treatment for PEH.

**Methods:**

Single center cohort analysis of all consecutive patients who underwent gastropexy for PEH without fundoplication. Postoperative outcomes and functional results were retrospectively collected. Reflux symptoms developed postoperatively were reported using the validated quality of life questionnaire: GERD-Health Related Quality of Life Qestionnaire (GERD-HRQL).

**Results:**

Thirty patients (median age: 72 years (65–80)) were included, 40% classified as ASA III. Main PEH symptoms were reflux (63%), abdominal/thoracic pain (47%), pyrosis (33%), anorexia (30%), and food blockage (26%). Twenty-six laparoscopies were performed (86%). Major complications (III-IVb) occurred in 9 patients (30%). Seven patients (23%) had PEH recurrence, all re-operated, performing a new gastropexy. Median follow-up was 38 (17–50) months. Twenty-two patients (75%) reported symptoms resolution with median GERD-HRQL scale of 4 (1–6). 72% (n = 21) reported operation satisfaction. GERD-HRQL was comparable between patients who were re-operated for recurrence and others: 5 (2–19) versus 3 (0–6), p = 0.100.

**Conclusion:**

Gastropexy without fundoplication was performed by laparoscopy in most cases with acceptable complications rates. Two-thirds of patients reported symptoms resolution, and long-term quality-of-live associated to reflux symptoms is good. Although the rate of PEH recurrence requiring a new re-intervention remained increased (23%), it does not seem to affect long-term functional results.

## Introduction

Paraoesophageal hernias (PEH) are associated with a high incidence of serious complications, such as gastrointestinal perforation, bleeding, volvulus with obstruction, or strangulation [1]. Symptomatic PEH often occurs in elderly and fragile patients after an asymptomatic course of mostly unknown duration [2]. Current guidelines from the Society for Endoscopic Gastrointestinal Surgery (SAGES) recommend the repair of all PEHs [3], especially in the setting of gastric volvulus or obstructive symptoms. Nevertheless, there is no consensus regarding the best surgical procedure for PEH repair [4].

Originally described in 1992, laparoscopic hiatal hernia repair with hiatoplasty (narrowing of the hiatus by suturing the diaphragmatic crura) involves circumferential hiatal dissection with excision of the hernia sac, mobilization of the esophagus and stomach, repositioning of both structures into the abdominal cavity, and fundoplication. This procedure has become the widely accepted intervention [5–7]. However, surgery remains technically difficult and delicate, particularly in fragile patients with large hiatal defects (gastro-thorax) and is almost exclusively performed in dedicated centers [8]. Furthermore, while fundoplication offers the advantage of preventing gastroesophageal reflux, it has an inherited risk of persistent dysphagia (up to 25%), as well as symptoms such as gas bloating and abdominal cramps [9].

Laparoscopic gastropexy without fundoplication, which involves attaching the stomach to the anterior abdominal wall to prevent mediastinal recurrences, is a valuable alternative procedure. Commonly, surgeons consider it as salvage procedure when formal repair of the paraesophageal hiatal hernia cannot be safely performed [10, 11] or for patients in poor general conditions and/or with multiple comorbidities [12]. In this approach, fundoplication, is not performed to avoid potential postoperative dysphagia, and to minimize the surgical trauma. The short- and long-term functional results after gastropexy without fundoplication are only scarcely described in the current literature. Our study aims to evaluate the short-term outcomes and long-term quality of life of patients who underwent hiatal repair and gastropexy alone as a treatment for PEH.

## Patients and methods

### Design of the study

A single-center retrospective cohort analysis was conducted, including all consecutive patients who underwent hiatal repair and gastropexy for PEH (Hill classification type II-IV) [13])without fundoplication from January 2015 to December 2021. The primary outcome was short-term postoperative complications, and secondary outcomes included functional results and long-term quality of life after gastropexy without fundoplication for PEH.

### Data collection and analysis

Patients' characteristics, perioperative data, and postoperative outcomes were retrospectively collected through chart review during routine follow-up. A dedicated database was created, capturing main demographic variables, surgical data, and postoperative complications according to the Clavien-Dindo classification [14]. Patients' reflux symptoms and quality of life were assessed via telephone interviews using the published and validated GERD-Health Related Quality of Life Questionnaire (GERD-HRQL) [15].

### Surgical technique

The surgical technique of hiatal hernia repair and gastropexy performed in our patients includes complete and systematic resection of the hernia sac, mobilization of the lower esophagus to achieve an intra-abdominal esophageal length of approximately 3 to 4 cm, ensuring the gastroesophageal junction is not pulled upward. Closure of the diaphragmatic hiatus is achieved with interrupted sutures of non-absorbable braided thread reinforced with surgical patches of polytetrafluoroethylene (PTFE “pledgets”) to support the suture and induce fibrosis. If necessary, a gastric calibration tube is used to exclude potential stenosis at the hiatus during closure. Gastropexy is typically performed with continuous suture between the fundus and the anterior abdominal wall using a non-absorbable, barbed suture.

### Participants and ethical approval

The study was conducted in accordance with the guidelines of the Declaration of Helsinki. Institutional General Consent for Research was obtained from all participants, and the study received approval from the Ethics Committee of Canton de Vaud (CER-VD), Lausanne, Switzerland: #2023–00417. Exclusion criteria included refusal or lack of Institutional General Consent for Research. The study also excluded underage patients.

### Statistical analysis

Continuous variables were presented as mean with standard deviation (SD) or median with interquartile range (IQR), depending on their distribution. Categorical variables were reported as frequencies (%) and compared using the chi-square test. Student's t-test or the Mann–Whitney test was used for comparing continuous variables. All statistical tests were two-sided, and a significance level of 0.05 was used. Statistical analyses were performed using GraphPad Prism 8 software (GraphPad Software, Inc., La Jolla, CA, USA).

## Results

Thirty patients (median age: 72 years (range: 65–80)) were included, with a median follow-up of 38 months (SD: 17–50). Due to the loss of follow-up for one patient, 29 patients (97%) completed the GERD-HRQL questionnaire. Forty-seven percent of the patients had thoracic or abdominal symptoms before surgery. Other main symptoms that led to surgery were food blockage, nausea/vomiting, and dyspnea. Four patients had a bleeding ulcer or gastric perforation as initial symptom. Nineteen patients (63%) experienced reflux symptoms before surgery. Eight surgeries (27%) were performed as emergencies, all for gastric perforations or signs of ischemic distress on the CT scan. Baseline and operative characteristics are described in Table 1. Major complications (III-IVb) occurred in 9 (30%). Seven patients (23%) had PEH recurrence and underwent redo surgery performing a new gastropexy. No patient died due to surgical complications. Post-operative outcomes are depicted in Table 2.

**Table 1:** Baseline and operative characteristics

Patients Characteristics	
Gender	
Male	13 (44%)
Female	17 (56%)
Age (median, years)	72 (65–80)
BMI (kg/m2)	26.5 (24–31)
ASA	
II	18 (60%)
III	12 (40%)
Comorbidities	
Congestive heart failure/Coronary disease	7 (23%)
COPD/ Pulmonary fibrosis	4 (13%)
Diabetes	2 (7%)
Symptoms of paraoesophageal hernia	
Vomiting/ anorexia	9 (30%)
Blockage	8 (26%)
Dyspnea	2 (7%)
Abdominal/ thoracic pain	14 (47%)
Bleeding ulcer/ gastric perforation	4 (13%)
Reflux symptoms before surgery	19 (63%)
**Peroperative Characteristics**	
Duration (minutes)	143 (114–180)
Emergency gastropexy	8 (27%)
Surgical approach	
Laparoscopy	26 (86%)
Laparotomy	2 (7%)
Conversion to laparotomy	2 (7%)
Additional procedure	8 (27%)
Umbilical hernia repair	2
Adhesiolysis (> 1 h)	2
Collis	2
Gastric wedge (for peroration)	1
Thoracic drain	2
Additional herniated organs	
Transverse colon	1 (3%)

*Data are presented as the number of patients (percentages), mean* ± *standard deviation, or median [25th; 75th percentiles]. Statistical significance (p* < *0.05). COPD: Chronic obstructive pulmonary disease*

**Table 2:** Post-operative outcomes

Post-operative outcomes	
Follow-up (months)	38 (17–50)
Postoperative complications	
Pneumonia	1 (3%)
Gastroparesis requiring NGT > 24 h	3 (10%)
Esophageal perforation	1 (3%)
Gastro-thorax recurrence (reoperation)	7 (23%)
Recurrence before 30 days	4 (13%)
Recurrence after 30 days	3 (10%)
Days to recurrence	28 (14–233)
Complication according to Clavien-Dindo [14]	15 (50%)
I-II	6 (20%)
III-IVb	9 (30%)
Length of hospital stay (days)	7 (5–10)

*Data are presented as the number of patients (percentages), mean* ± *standard deviation, or median [25th; 75th percentiles]. Statistical significance (p* < *0.05). NGT: Nasogastric tube*

**Fig. 1:**
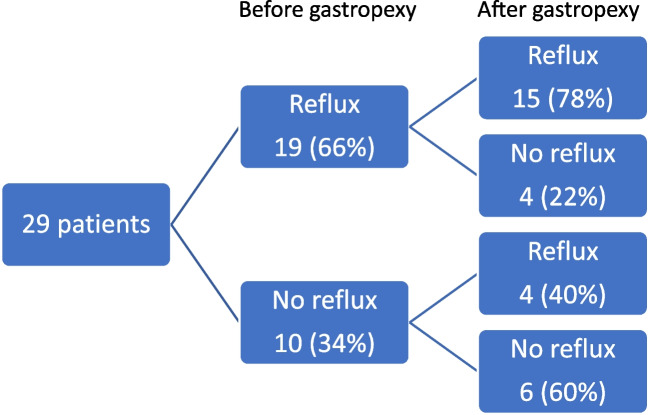
Patients flow chart of reflux symptoms before and after gastropexy (*n *= 29 patients)

During follow-up, 21 (75%) reported symptoms resolution with median GERD-HRQL scale of 4 (1–6) (best possible score: 0, worst possible score: 50). Seventy-two percent (*n* = 21) reported operation satisfaction. GERD-HRQL was comparable between patients who were re-operated for recurrence and others: 5 (2–19) versus 3 (0–6), *p* = 0.100. Functional results and long-term quality of life are described in Table 3. After gastropexy four patients develop a reflux de novo and another four had complete reflux resolution after gastropexy as showed in patients flow-chart (Fig. 1).

**Table 3:** Symptoms outcomes stratified by recurrence (n = 29 patients with GERD-HRQL)

Patients’ symptoms outcomes (n = 29)	No recurrence n = 22	Recurrence n = 7	p-value
GERD-HRQL scale	3 (0–6)	5 (2–19)	0.100
Preoperative symptoms improvement			0.002
Complete resolution	9 (42%)	1 (14%)	
Significant	11 (50%)	1 (14%)	
Minimal	1 (4%)	5 (72%)	
No improvement	1 (4%)	0	
Requiring medications			0.063
None	10 (45%)	0	
Proton-pump inhibitors	12 (55%)	7 (100%)	
Dietary restriction due to symptoms	8 (36%)	3 (42%)	
Satisfaction with present condition			0.011
Satisfied	19 (87%)	2 (29%)	
Neutral	2 (9%)	4 (57%)	
Dissatisfied	1 (4%)	1 (14%)	
“If you have to do it again?”	20 (90%)	5 (71%)	0.192

*Data are presented as the number of patients (percentages), mean* ± *standard deviation, or median [25th; 75th percentiles]. Statistical significance (p* < *0.05). GERD-HRQL scale *[15]*: Gastroesophageal Reflux Disease-Health-Related Quality of Life scale*

## Discussion

This series assessed the outcome of 30 patients who underwent laparoscopic gastropexy without fundoplication for PEH. One-third of the patients experienced postoperative complications classified as grade III or higher. Approximately two-thirds of the patients experienced resolution of their symptoms, and the long-term quality of life related to reflux symptoms was found to be satisfactory. Although there was a high rate of recurrence requiring re-intervention, this did not appear to have a negative impact on long-term functional outcomes.

Gastropexy without fundoplication has been shown to be an effective surgical alternative for patients with comorbidities, offering a shorter and less invasive procedure with reduced surgical risk [7, 10, 12]. To date, only one published study in 2020 [7] has examined the long-term outcomes of laparoscopic gastropexy without fundoplication in 26 patients, with a follow-up of 28 months. In this study, a postoperative complication rate of 35% was observed, with 8% of patients requiring reintervention. However, 88% of patients reported significant improvement or complete resolution of their symptoms, and all expressed satisfaction with their current health status, although the majority continued to take proton pump inhibitors [7]. These findings are consistent with our observations. Indeed, the majority of patients were satisfied with the procedure, reporting significant improvement in their symptoms. Furthermore, no significant difference was observed between patients who required reintervention and those without early recurrence in terms of reported symptoms and quality of life. Another prospective study published by Daigle et al. [16] followed a total of 101 patients (follow-up duration: 10.8 months) who underwent anterior gastropexy without fundoplication for PEH. This study reported a recurrence rate of 17%, but 70% of patients reported being symptom-free from reflux. This study mainly focuses on symptom improvement after laparoscopic gastropexy, but the follow-up periods are relatively short and without considering quality of life-related outcomes.

When performing surgery for PEH without creating an anti-reflux valve, the risk of developing or worsening gastroesophageal reflux is a major concern [17]. However, the results of our study indicate that this risk is not observed. It is important to note that fundoplication, which involves creating an anti-reflux valve, is a major intervention for frail and elderly patients [1]. Moreover, post-fundoplication dysphagia can occur in up to 25% of patients [9], with a risk of gastroesophageal reflux recurrence of up to 17% [18]. Risk factors such as advanced age and comorbidities were identified to contribute to these outcomes [1].

Due to the presence of a large PEH it can be extremely difficult, if not impossible, to perform preoperative manometry and impedance pH monitoring, which are typically recommended, especially in cases of extra-digestive manifestations [3]. This information could be crucial for guiding the choice of surgical treatment and improving intervention outcomes.

There is no standardized method for gastric fixation to the abdominal wall regarding gastropexy [7]. Our technique involves laparoscopic continuous suturing using a preferably a non-absorbable unidirectional barbed monofilament suture. Other methods such as distal anterior gastropexy or percutaneous gastrostomy have been suggested, but their short- and long-term results have not been published on a series of patients [19–22].

We observed that nearly one-third of the patients experienced major complications. This finding highlights the high risk associated with surgery for hiatal hernia, further reinforcing our conviction regarding the prudence of performing gastropexy instead of extensive repair with fundoplication. Our results suggest that these patients can expect to have a good quality of life even without the creation of an anti-reflux valve. During the observation period of our series, all patients who underwent emergency surgery for a paraoesophageal hernia (27%) received gastropexy without fundoplication. This surgical approach can be particularly beneficial for surgeons with limited experience in functional surgery, who may encounter these patients in emergency situations.

Our retrospective study has several limitations that should be mentioned. We do not have data on preoperative quality of life using GERD-HRQL in our cohort, limiting our ability to assess the change in quality of life conferred by the operation. However, by analyzing medical records, we were able to identify preoperative symptoms related to PEH and compare them to postoperative symptoms. The small size of our population should also be considered a limitation in its interpretation. Nevertheless, the response rate was high, approximately 97%. It is challenging to obtain a substantial population for the study of this procedure as its necessity is relatively rare. We believe that further studies involving a larger sample of patients, particularly elderly individuals with comorbidities, are needed to confirm that gastropexy could be the intervention of choice for repairing PEH in this patient group.

## Conclusion

Gastropexy without fundoplication was performed by laparoscopy in most cases with acceptable complications rates. Two-thirds of patient’s report symptoms resolution, and long-term quality-of-live associated to reflux symptoms is good. Although the rate of PEH recurrence requiring a new re-intervention remains increased (23%), it does not seem to affect long-term functional results. This technique appears to be a valid alternative in emergency situations, as well as for symptomatic and fragile patients with comorbidities, where preoperative assessment of reflux disease is often unavailable or challenging to conduct. Moreover, for surgeons less experienced in functional surgery, this approach could represent a valuable alternative in emergency situations where anti-reflux fundoplication is not feasible.

## Data Availability

Data are not available to protect study participant privacity in accordance with the recommendations of the local ethics committee.
